# A Proactive Approach for Managing COVID-19: The Importance of Understanding the Motivational Roots of Vaccination Hesitancy for SARS-CoV2

**DOI:** 10.3389/fpsyg.2020.575950

**Published:** 2020-10-19

**Authors:** Steven Taylor, Caeleigh A. Landry, Michelle M. Paluszek, Rosalind Groenewoud, Geoffrey S. Rachor, Gordon J. G. Asmundson

**Affiliations:** ^1^Department of Psychiatry, The University of British Columbia, Vancouver, BC, Canada; ^2^Department of Psychology, University of Regina, Regina, SK, Canada

**Keywords:** COVID-19, SARSCoV2, pandemic, coronavirus, vaccination, vaccination hesitancy, vaccination attitudes

## Abstract

**Importance:**

Vaccination hesitancy—the reluctance or refusal to be vaccinated—is a leading global health threat (World Health Organization, 2019). It is imperative to identify the prevalence of vaccination hesitancy for SARS-CoV2 in order to understand the scope of the problem and to identify its motivational roots in order to proactively prepare to address the problem when a vaccine eventually becomes available.

**Objective:**

To identify (1) the prevalence of vaccination hesitancy for a SARS-CoV2 vaccine, (2) the motivational roots of this hesitancy, and (3) the most promising incentives for improving the likelihood of vaccination uptake when a vaccine does become available.

**Design, Setting, and Participants:**

A cross-sectional sample of 3,674 American and Canadian adults assessed during the COVID-19 pandemic in May 2020.

**Main Outcomes:**

Measures of vaccination intention (i.e., “If a vaccine for COVID-19 was available, would you get vaccinated?”), attitudes toward vaccines in general and specific to SARS-CoV2 using the Vaccination Attitudes Examination Scale, and incentives for getting vaccinated for those who reported they would not get vaccinated.

**Results:**

Many American (25%) and Canadian (20%) respondents said that they would not get vaccinated against SARS-CoV2 if a vaccine was available. Non-adherence rates of this magnitude would make it difficult or impossible to achieve herd immunity. Vaccine rejection was most strongly correlated with mistrust of vaccine benefit, and also correlated with worry about unforeseen future effects, concerns about commercial profiteering from pharmaceutical companies, and preferences for natural immunity. When asked about incentives for getting vaccinated, respondents were most likely to report that evidence for rigorous testing and safety of the vaccine were of greatest importance.

**Conclusions and Relevance:**

Vaccination hesitancy is a major looming problem for COVID-19. To improve vaccine uptake, it is imperative that the vaccine is demonstrated to the public to be rigorously tested and not perceived as rushed or premature in its dissemination.

## Introduction

Vaccination hesitancy—the reluctance or refusal to be vaccinated—is among the top ten global health threats (World Health Organization, 2019). It is a major problem for seasonal influenza (World Health Organization, 2019), was a significant problem during the 2009 H1N1 pandemic (Bangerter et al., 2012), and may be increasing in recent years (Yaqub et al., 2014). Vaccination hesitancy is also a growing problem among healthcare workers (Maltezou et al., 2018). In order to proactively manage the COVID-19 pandemic, it is important to identify the prevalence of vaccination hesitancy for SARS-CoV2. A review of 12 studies found that the mean R_0_ for COVID-19 virus is 3.28 (Liu et al., 2020), suggesting that the threshold for achieving herd immunity (1–1/R_0_) is 70% (Fine et al., 2011). Since people who refuse vaccination are not randomly dispersed (i.e., they tend to occur in clusters) (Fine et al., 2011), more than 70% of people in a community would need to be vaccinated in order to achieve herd immunity.

Mandatory vaccination is unlikely to be a viable option in individualistic societies due to increasing anti-vaccination sentiment (Taylor, 2019). If vaccination hesitancy for SARS-CoV2 is prevalent, then it is important to identify the motivational roots (i.e., attitudes or reasons) underlying the reluctance (Hornsey et al., 2018) and ways to address these. Public education programs (e.g., “do it for the herd”) can be helpful to some extent (Taylor, 2019); but, identifying motivational factors for vaccination hesitancy and then proactively tailoring public health messaging and incentives to address these factors prior to beginning an immunization program may improve overall vaccine uptake (World Health Organization, 2020).

The purpose of this study was to assess a population-representative sample of adults from the United States and Canada in order to identify (1) the prevalence of vaccination hesitancy for a SARS-CoV2 vaccine when one does become available, (2) the motivational roots of this hesitancy, and (3) the most promising incentives for improving the likelihood of vaccination uptake when a vaccine does become available.

## Materials and Methods

### Design

A cross-sectional design was used in which participants completed an internet-based battery of questionnaires, including demographic questions and measures of vaccination intention, attitudes, and incentives.

### Sample and Data Collection Procedures

Data were collected from May 6–19, 2020, from 3,674 adults recruited from communities in the United States (*n* = 1,772) and Canada (*n* = 1,902) using an internet-based self-report survey delivered in English by Qualtrics, which is a commercial survey sampling and administration company. Qualtrics maintains a pool of potential participants who have agreed to be contacted in order to respond to surveys. For the present study, Qualtrics selected and contacted participants to meet sampling quotas based on age, gender, ethnicity, socioeconomic status, and geographic region within each country to obtain a representative sample. Filters were used to eliminate data from careless or incomplete responses. Respondents received credit points for participation, similar to points in a credit card rewards program, which could be converted into currency. For the present study, respondents received credit points equivalent to US$7.00 for participating in the online study. All respondents provided written informed consent prior to completing the survey. All procedures followed were in accordance with the standards of the Helsinki Declaration. The research described in this article was approved by the Research Ethics Board of the University of Regina (REB# 2020-043).

### Measures

Vaccination attitudes were measured using two versions of the Vaccination Attitudes Examination Scale (Martin and Petrie, 2017), assessing general vaccination attitudes and attitudes specific to SARS-CoV2. Both versions contain four subscales, including mistrust of vaccine benefit, worries over unforeseen future effects of the vaccine, concerns about commercial profiteering from the vaccine, and preference for natural immunity.

Regarding the assessment of vaccine uptake, vaccination is a binary event (i.e., a person does or does not get vaccinated); accordingly, participants answered a forced-choice yes/no question to measure vaccination intention: “If a vaccine for COVID-19 was available, would you get vaccinated?” A “don’t know” or “uncertain” response option was omitted because it simply defers endorsing a decision.

Incentives for getting vaccinated were assessed only for people who responded “no” to the measure of vaccination intention (incentives were not assessed for “yes” responders because they were not in need of additional vaccination incentives). “No” responders were presented a list of 21 incentives (Table 1) and asked to rate whether each would increase their chances of getting vaccinated using a 5-point scale (0 = definitely would not, 4 = definitely would). The relative efficacy of each incentive was calculated by computing the percentage of respondents who gave a rating of 3 or 4 (“probably would” or “definitely would”).

**TABLE 1:** Point-biserial correlations between the decision to not get vaccinated against SARS-CoV2 and negative attitudes about a SARS-CoV2 vaccine and vaccines in general.

Type of negative attitude	Concerning SARS-CoV2 vaccine	Concerning vaccines in general
Mistrust of vaccine benefit	0.64*	0.42*
Worry about unforeseen future negative effects	0.33*	0.33*
Concerns about commercial profiteering	0.43*	0.37*
Preference for natural immunity	0.43*	0.30*

*p < 0.001.

## Results

A total of 43% of the sample were female, most (92%) were employed full- or part-time, and most (82%) had completed full or partial college. Most (69%) were Caucasian, with the remainder being Asian (12%), African American/Black (9%), Latino/Hispanic (6%), or other (5%). Only 2% of the sample reported being diagnosed with COVID-19, and only 3% were healthcare workers who might come into contact with patients infected with SARS-CoV2. Sample mean age was 53 years (SD = 15 years, range 18–94 years). According to American and Canadian census records, the population mean age (including children and adults) is approximately 40 years (Statistics Canada, 2020; United States Census Bureau, 2020). The mean age of sample is what would be expected from a population representative sample consisting only of adults.

In response to the question of whether participants would get vaccinated against SARS-CoV2, if a vaccine was available, 25% of Americans and 20% of Canadians said “no.” Significantly more Americans than Canadians said that they would not get vaccinated, χ^2^(*df* = 1) = 12.41, *p* < 0.001. Table 1 shows the correlations between vaccination intention (1 = no, 0 = yes) and negative attitudes toward vaccination. All of the negative attitudes toward a SARS-CoV2 vaccination, and vaccinations in general, were significantly correlated (*p* < 0.001) with the decision to not get vaccinated against SARS-CoV2. The largest correlation was between “no” to vaccination and mistrust of the benefit of a SARS-CoV2 vaccine. This correlation was significantly larger than all of the other correlations in Table 1 (*p* < 0.001). These correlations were moderate-to-large in magnitude (rs > 0.30), according to Cohen’s classification (Cohen, 1988).

According to Cohen’s classification, correlations of 0.20 are considered small. Given the sample size, trivially small correlations (<0.20) were statistically significant. This was the case concerning the correlation between vaccination refusal and demographic variables, which were statistically significant but trivial in magnitude: Female gender *r* = 0.10, *p* < 0.001; age *r* = 0.11, *p* < 0.001; completed full or partial college education (vs. did not complete) *r* = 0.10, *p* < 0.001, unemployed r = −0.050, *p* < 0.005, minority status (vs. Caucasian) *r* = −0.04, *p* < 0.05.

For respondents indicating they would not get vaccinated against SARS-CoV2, Table 2 shows the percentage who probably would/definitely would get vaccinated if incentives were provided. Consistent with the finding that the strongest correlate of vaccination refusal was concern about the benefit of the vaccine, the most efficacious incentives were those providing evidence that the vaccine was safe and efficacious. In other words, the most efficacious incentives were those that matched the motivational roots of vaccination hesitancy for a SARS-CoV2 vaccine. The least efficacious incentives involved promotions for vaccine uptake from social media, news media, or from community leaders.

**TABLE 2:** Respondents stating that they would *not* get vaccinated against SARS-CoV2 (*n* = 812): Percentage reporting that the following incentives would probably or definitely induce them to get vaccinated.

Incentive	% Probably or definitely would get vaccinated
If I was convinced that the vaccine had been rigorously tested	38
If I saw that enough people were safely vaccinated without negative side effects	36
If I saw that enough people who got the vaccine didn’t get sick with COVID-19	34
If I saw that my friends and family didn’t have negative side effects from the vaccine	34
If getting vaccinated was a requirement for my job	31
If I thought the health authorities were trustworthy	29
If I was convinced that getting vaccinated helped protect vulnerable members of my community	25
If getting vaccinated was required by my government	25
If a trusted health care worker told me to get vaccinated	22
If I knew that I was not being exploited by the pharmaceutical industry	19
If getting vaccinated was required for me to attend social or sporting events	19
If someone I knew died from COVID-19	18
I received a financial incentive	18
If I was assured that the government wasn’t controlling the vaccine	17
If someone I knew got sick with COVID-19	16
If someone I knew was hospitalized because of COVID-19	16
If I received some other incentive (e.g., discount coupon)	8
If a news source that I trust promoted vaccination	8
If religious leaders in my community said I should get vaccinated	6
If my President or Prime Minister promoted the vaccine	6
If vaccination was promoted in my social media network	4

## Discussion

Anticipating and preparing for problems concerning vaccination adherence when a vaccine for SARS-CoV2 becomes available is a critical step in managing the COVID-19 pandemic. Research suggests that greater than 70% of the population will need to be vaccinated against SARS-CoV2 to achieve herd immunity (Fine et al., 2011). Our research suggests that 25% of Americans and 20% of Canadians would reject a SARS-CoV2 vaccine, raising concerns that herd immunity might not be attained when a vaccine becomes available.

The degree of vaccination hesitancy found in the present study is broadly consistent with other studies that were published after our study had been completed. Studies conducted during March–April 2020, which was somewhat earlier than the present study (May, 2020), reported findings broadly similar to ours in terms of percentages of people who stated that they would not get vaccinated against SARS-CoV2: Italy (14%) (Barello et al., 2020), France (26%) (COCONEL Group, 2020), and Australia (14%) (Dodd et al., 2020). In a European survey in June, 2020, 24% of respondents stated that they were either unwilling or unsure about getting vaccinated (Neumann-Böhme et al., 2020). More recent surveys (August–September, 2020) in the United States and Britain suggest that upward of 50% of people would not get vaccinated (Bracken, 2020; McKie, 2020). Thus, vaccination hesitancy is an important and possibly growing problem.

Another concerning finding from the present study is that rejection of a SARS-CoV2 vaccine was associated with negative attitudes toward vaccination in general. If SARS-CoV2 persists during the forthcoming influenza season, then people might need to be vaccinated against both SARS-CoV2 and seasonal influenza. There could be devastating consequences, with widespread seasonal infection of both viruses, if people with negative attitudes about vaccination reject both vaccines. In our study, rejection of vaccination against SARS-CoV2 was correlated with a range of negative attitudes about a SARS-CoV2 vaccine, and vaccines in general, with the strongest correlation regarding mistrust about the benefits of a SARS-CoV2 vaccine. The public has been exposed to false hopes about COVID-19 treatments, such as the use of hydroxychloroquine (U.S. Food & Drug Administration, 2020), undermining confidence in the recommendations of community leaders. Our research suggests that exposure to authoritative information is a stronger incentive for vaccination than mere endorsements from community leaders or social media influencers.

Consistent with the present study, other studies appearing after our study had been completed have found that vaccination hesitancy is associated with negative attitudes toward a SARSCoV2 vaccine, including concerns about safety and efficacy (Fisher et al., 2020; Neumann-Böhme et al., 2020; Palamenghi et al., 2020), and doubts about the necessity for vaccination (Dodd et al., 2020). Findings from the present study, along with results from previous studies, have important implications for public policy. In order to maximize the uptake of a SARSCoV2 vaccine, when such vaccine becomes available, it is important to address the various anti-vaccination beliefs identified in the present study and in other recent investigations. Across studies, a commonly identified concern is that the risks might outweigh the benefits. Our research found that participants would be more likely to get vaccinated if they were persuaded that the vaccine had been rigorously tested (Table 2). In order to maximize vaccine uptake, health authorities need to reassure the public that vaccine development has followed all the preestablished guidelines and that the process of developing a vaccine has not been rushed. If the public perceives that health authorities are hastily rushing a SARS-CoV2 vaccine into production, then this would undermine public confidence and exacerbate vaccination refusal. Our findings suggest that the most important way of ensuring vaccine uptake is to provide the public with convincing evidence that a SARS-CoV2 vaccine has been rigorously tested, shown to be effective, and is not perceived as being rushed into production. Unfortunately, the vaccine production program by the U.S. Department of Health and Human Services is called “Operation Warp Speed”^1^. For people in the community who are worried that the vaccine production process has been excessively rushed, the name “Operation Warp Speed” sends a disturbing message; it suggests that due diligence has not been followed and that there has not been sufficient evaluation of the comparative risks and benefits. Mistrust of health authorities is an important deterrent to vaccination uptake (Taylor, 2019). Vaccination development and dissemination programs with more reassuring titles would be more likely to engage the public trust (e.g., calling the program “Operation Due Diligence” instead of “Operation Warp Speed”).

The present study has various strengths and limitations. In terms of strengths, the sample was large and the study provides new information on barriers and incentives for people to get vaccinated against SARSCoV2. A limitation of the study is the cross-sectional nature of the design. It is possible that COVID-19-related vaccination attitudes may change over time, especially if governments or health authorities launch pro-vaccination public education programs. This remains to be investigated in future research. The question of whether vaccination attitudes differ across different ethnic or cultural groups also remains to be investigated. Additional research is also needed to investigate whether variables other than those investigated in the present study are association with vaccination hesitancy. Such variables might include health literacy and other individual difference variables. The question of whether the findings of the present study can be generalized across different countries and cultures also remains to be investigated.

Another limitation of this study is that political affiliation was not measured. Other surveys suggest that people who oppose a SARS-CoV2 vaccine are more likely have Conservative or Republican political affiliations than Liberal or Democrat affiliations in both the United States and Canada (Angus Reid Institute, 2020; Gallup, 2020). A further limitation is that we assessed vaccinations intentions rather than actual vaccination behaviors. This was unavoidable as a vaccine for SARS-CoV2 was not available at the time of this study. The study was conducted under the premise that it is more important to be proactive in addressing forthcoming vaccination problems than to be reactive in an attempt to deal with problems as they arise.

## Data Availability Statement

The raw data supporting the conclusions of this article will be made available by the authors, without undue reservation.

## Ethics Statement

The studies involving human participants were reviewed and approved by University of Regina Research Ethics Board. The patients/participants provided their written informed consent to participate in this study.

## Author Contributions

ST and GA contributed equally to this article in terms of study conceptualization, design, and procurement of funding. CL and MP organized data collection. RG contributed to the development of the assessment materials. ST analyzed the data. All authors contributed to the writing of the manuscript.

## Conflict of Interest

The authors declare that the research was conducted in the absence of any commercial or financial relationships that could be construed as a potential conflict of interest.

## Footnotes

1 https://www.hhs.gov/coronavirus/explaining-operation-warp-speed/index.html, accessed September 19, 2020.
